# Revisiting the De Facto Mental Health System: One Becomes Two

**DOI:** 10.1111/acem.70227

**Published:** 2026-01-22

**Authors:** Kurt Kroenke, Paul I. Musey, Jill D. Connors, Patrick O. Monahan

**Affiliations:** ^1^ Department of Medicine Indiana University School of Medicine and Regenstrief Institute Indianapolis Indiana USA; ^2^ Department of Emergency Medicine Indiana University School of Medicine Indianapolis Indiana USA; ^3^ Department of Surgery Indiana University School of Medicine Indianapolis Indiana USA; ^4^ Department of Biostatistics and Health Data Science Indiana University School of Medicine Indianapolis Indiana USA

In a classic 1978 study, Regier et al. [1] reported that three times as many individuals with a mental health (MH) condition received treatment in primary care as in the specialty mental health sector. Denoted by the authors as the “de facto mental health system,”, this disproportionate responsibility of primary care to detect and treat MH conditions was reaffirmed in a 2014 study by Olfson et al. [2] The emergency department has increasingly become a second de facto setting for MH care [3, 4, 5, 6]. Attention has often focused on psychiatric emergencies (suicidality, overdose, psychosis), whereas mood, anxiety, and substance use are, in fact, more prevalent in the ED setting [4] and often are the underlying or at least comorbid reason for seeking ED care, or they complicate the non‐MH condition.

## 1 Comparing the Two De Facto Mental Health Systems

The inordinate roles of primary care and the ED in MH care share some root causes: a shortage of mental health specialists, reimbursement policies that place MH care at a disadvantage to coverage of medical and surgical services, and public stigma about seeing an MH specialist. Also, somatic symptoms and medical disorders prompting persons to seek medical or emergency care can mask underlying psychological factors.

However, several factors may be contributing to a shift of some de facto MH care from primary care to the ED. Shortages in the primary care workforce parallel those in the MH specialty workforce: lower clinician reimbursement and lower prestige have made selection of either career less popular among medical students than other medical or surgical specialties. Thus, a number of persons in the United States may not have a primary care clinician or, even if they do, have difficulty getting an expedited appointment for new problems or concerns because of large panel sizes and appointments booked months in advance for the care and follow‐up of chronic medical problems. In this case, the default option for new problems is all too often going to the ED or urgent care. Unfortunately, the latter settings are best suited for the care of acute problems and not longitudinal follow‐up of MH problems, for which treatment often needs to be monitored and adjusted over months. Thus, the ED/urgent care setting is designed for episode‐based acute care, whereas the primary care setting can provide longitudinal chronic care.

## 2 Pat Solutions

The adjective *pat* has two (and opposing) definitions: (1) exactly suitable or opportune; (2) overly simplistic or too good to be true. Indeed, the common solutions below are pat in both senses: suitable if they can be executed, but often not feasible or ineffective unless linked to system changes, such as additional personnel or extra reimbursable time. They are good in principle but all too often not easy to implement in busy clinical practice settings.

1. *Educating providers*. Programs to improve the knowledge, skills, and attitudes of frontline clinicians are often proposed as an obvious and purportedly easy way of addressing MH disorders in ED or other frontline settings like primary care. The belief is that educated providers will do the “right thing.” However, continuing education alone in the absence of additional time or workflow‐enhancing resources is often insufficient to empower busy frontline providers with many competing demands.
2. *Screening for MH disorders*. Using brief scales to detect common disorders such as depression, anxiety, and substance use is another seemingly self‐evident step that, in essence, is conceived as a “free” lab test. The misconception is that finding it will lead to fixing it. Instead, most evidence suggests that screening should be coupled with system‐based changes to optimize depression treatment and monitoring to improve outcomes [7]. Like education, screening alone is unlikely to have major benefits in improving MH outcomes in the ED.
3. *Reattributing symptoms to psychological factors*. Patients with underlying MH disorders often present with somatic symptoms mimicking medical conditions (e.g., low‐risk chest pain caused by anxiety or headache or other pain aggravated by stress). However, simply telling patients presenting with noncardiac chest pain that their symptoms may be due to anxiety can prompt a knee‐jerk reaction, “You're telling me this is all in my head!” Ferreting out the potential link between psychological factors and somatic symptoms is not a rapid process but more often requires longitudinal follow‐up, where a deeper understanding of the patient and the building of trust are facilitated.
4. *Referring to specialty mental health*. This would be an ideal approach if there were not countervailing barriers. First, the mental health workforce is inadequate to meet the MH needs of the population; ED referrals pour more water into an overflowing bucket. Second, coverage and reimbursement issues specific to mental health further diminish ready access. Third, patients who are referred to mental health frequently do not make or keep their appointments. The Field of Dreams hope (“if you build it, they will come”) is often frustrated. Enhancing referral success may require some elements of care coordination, such as efforts to destigmatize MH treatment, identification of an MH provider, and scheduling an MH appointment, followed by reminders.
5. *Follow up with primary care*. Not all common mental disorders need a MH referral; indeed, many uncomplicated cases of depression and anxiety can be initially (and sometimes fully) managed in primary care. But advice from the ED to “follow up with your primary care provider” needs to recognize that many patients may not have a primary care provider, or those that do may find it difficult to get an expedited appointment. Also, over‐reliance on the primary care system may reduce the de facto role of the ED in MH care by shifting it to the second de facto system of primary care.

## 3 Moving Forward

The irony of the pat solutions is that while often insufficient as individual components, their collective use may be more effective. Indeed, several systematic reviews have found effectiveness for multi‐component interventions [8, 9]. ED‐based care plans and case management have proven an effective strategy for transition to outpatient MH care [10]. Whereas the ED will continue to have a pivotal role in psychiatric crises, its preferred role regarding patients presenting with nonurgent MH conditions is to triage and coordinate with mental health and/or primary care. In patients or settings where follow‐up with mental health or primary care may be considerably delayed, virtual interventions using remote monitoring of symptoms, self‐management programs, linkage to community resources, and/or telephone follow‐up with ancillary personnel (peers, health coaches) may be helpful in bridging the gap in transition [11, 12, 13]. Additionally, communication strategies to mitigate the common experience of patients with MH conditions feeling stigmatized and invalidated [14, 15, 16] might not only improve the experience in the ED but also facilitate subsequent acceptance of MH treatments. Providing some low level of resources for the pat solutions, plus combining and coordinating their use, can convert the first meaning of pat (overly simplistic) to its second meaning (suitable and well‐timed).

## Data Availability

The authors have nothing to report.
